# Technology-enabled activation of skin cancer screening for hematopoietic cell transplantation survivors and their primary care providers (TEACH)

**DOI:** 10.1186/s12885-020-07232-2

**Published:** 2020-08-03

**Authors:** Saro H. Armenian, Lanie Lindenfeld, Aleksi Iukuridze, Meagan Echevarria, Samantha Bebel, Catherine Coleman, Ryotaro Nakamura, Farah Abdullah, Badri Modi, Kevin C. Oeffinger, Karen M. Emmons, Ashfaq A. Marghoob, Alan C. Geller

**Affiliations:** 1 Department of Population Sciences, City of Hope, 1500, East Duarte Road, Duarte, CA 91010-3000 USA; 2 Department of Population Sciences, Dana-Farber Cancer Institute, Boston, MA USA; 3 Department of Hematology & Hematopoietic Cell Transplantation, City of Hope, Duarte, CA USA; 4 Department of Surgery, City of Hope, Duarte, CA USA; 5 Department of Medicine, Community and Family Medicine and Population Health Sciences, Duke Cancer Institution, Duke, North Carolina USA; 6 Department of Social and Behavioral Sciences, Harvard TH Chan School of Public Health, Boston, MA USA; 7 Department of Dermatology, Memorial-Sloan Kettering Cancer Center, New York, NY USA

**Keywords:** Hematopoietic cell transplantation, Survivors, Skin cancer, Skin self-examination, Dermoscopy, Early detection, Patient activation

## Abstract

**Background:**

Hematopoietic cell transplantation (HCT) is a curative option for a growing number of patients with hematologic diseases and malignancies. However, HCT-related factors, such as total body irradiation used for conditioning, graft-versus-host disease, and prolonged exposure to immunosuppressive therapy, result in very high risk for melanoma and non-melanoma skin cancer (NMSC). In fact, skin cancer is the most common subsequent neoplasm in HCT survivors, tending to develop at a time when survivors’ follow-up care has largely transitioned to the primary care setting. The goal of this study is to increase skin cancer screening rates among HCT survivors through patient-directed activation alone or in combination with physician-directed activation. The proposed intervention will identify facilitators of and barriers to risk-based screening in this population and help reduce the burden of cancer-related morbidity after HCT.

**Methods/design:**

720 HCT survivors will be enrolled in this 12-month randomized controlled trial. This study uses a comparative effectiveness design comparing (1) *patient activation and education* (PAE, *N* = 360) including text messaging and print materials to encourage and motivate skin examinations; (2) *PAE plus primary care physician activation* (PAE + Phys, N = 360) adding print materials for the physician on the HCT survivors’ increased risk of skin cancer and importance of conducting a full-body skin exam. Patients on the PAE + Phys arm will be further randomized 1:1 to the *teledermoscopy* (PAE + Phys+TD) adding physician receipt of a portable dermatoscope to upload images of suspect lesions for review by the study dermatologist and an online course with descriptions of dermoscopic images for skin cancers.

**Discussion:**

When completed, this study will provide much-needed information regarding strategies to improve skin cancer detection in other high-risk (e.g. radiation-exposed) cancer survivor populations, and to facilitate screening and management of other late effects (e.g. cardiovascular, endocrine) in HCT survivors.

**Trial registration:**

ClinicalTrials.gov, NCT04358276. Registered 24 April 2020.

## Background

### Melanoma/skin cancer in the general population

Skin cancer, including melanoma and non-melanoma (NMSC), is diagnosed in 1 in 5 Americans during their lifetime and is the most common cause of cancer in the United States (U.S) [1]. Although the risk of dying from skin cancer is lower than many other cancers, the management of skin cancer is not without physical, psychological, and financial burden, with annual financial costs for patients alone estimated at more than $8 billion per year [1]. The incidence of melanoma, the most commonly fatal type of skin cancer, continues to rise [2]; the true incidence of less-lethal NMSCs, such as basal cell carcinoma (BCC) and squamous cell carcinoma (SCC), is difficult to enumerate, as they are not tracked in national databases [2].

The Surgeon General wrote a Call to Action to Prevent Skin Cancer in 2014 [3], emphasizing skin cancer as a major public health concern. The U.S. Preventive Services Task Force (USPSTF) recently concluded that there was insufficient evidence to recommend screening for skin cancer in the general population, due to concern about potential harms, including the psychological stress of screening [4]. However, the USPSTF also emphasized that future research on skin cancer screening should focus on evaluating the effectiveness of targeted screening in people considered to be at higher risk for skin cancer, while measuring the possible benefits and harms of screening by both at-risk individuals and their providers [4].

### The high burden of health complications, including skin cancer, among HCT survivors

Hematopoietic cell transplantation (HCT) is a curative option for a growing number of patients with hematologic diseases and malignancies. By the year 2030, there will be more than 500,000 HCT survivors in the U.S. [5] These survivors have been exposed to chemotherapy, other immunosuppressive therapy, and/or radiation before HCT (for management of primary cancer), at time of HCT (for the transplant procedure), and after HCT (for graft-versus-host disease [GVHD] and/or relapse of primary cancer) [5, 6]. Cumulative therapeutic exposures injure normal tissues, leading to premature onset of chronic health conditions such as subsequent cancers [6, 7]. We have shown that HCT survivors have a greater than 10-fold risk of developing subsequent cancers after HCT [8, 9], with skin cancer accounting for up to 60% of all subsequent solid cancers [8, 10]. Treatment-related risk factors for skin cancer after HCT include radiation therapy (melanoma, BCC), chronic GVHD (SCC), length and severity of immunosuppression (SCC), and prolonged exposure to the anti-fungal voriconazole (SCC) [11–15]; it is estimated that > 80% of all HCT survivors will have at least one treatment-related risk factor for skin cancer [12]. In these survivors, the risk of melanoma is greater than five-fold higher (Hazard Ratio [HR]: 5.5, 95% CI, 1.7–17.7) than in the general population [16], and the incidence of BCC and SCC exceeds 10% at 10 years after HCT [11, 16]. The epidemiology of skin cancer in HCT survivors also differs from that of the general population, as survivors develop skin cancers at a younger age, present with advanced disease, and are more likely to have multiple recurrences, with a shorter latency between recurrences [11, 12, 17, 18]. Due to anatomic location, some skin cancers are more visible to the patient, whereas others require discovery by a physician or family member, emphasizing the need for team-based approaches for monitoring and early detection of skin cancer in these individuals.

### The importance of skin examinations

Because skin cancer and its precursors can be easily seen by the patient and caregivers, teaching skin self-examination (SSE) and encouraging them to alert primary care physicians (PCPs) to skin changes is a key opportunity for health promotion. At-risk individuals (e.g. family history of skin cancer, prolonged sun exposure) should be encouraged to perform regular SSE (i.e. monthly) and should be educated as to the signs of suspicious pigmented lesions using the American Academy of Dermatology ABCDE (Asymmetry, Border, Color, Diameter, Evolution) algorithm [19]. Indeed, performing SSE may reduce mortality from melanoma by up to 63% [20]. Two studies in the general population provide the strongest evidence to date for the benefits of physician skin screening, showing 32% increased odds of having a smaller (i.e. more curable) melanoma at diagnosis following physician screening, and 40% reduction in melanoma mortality for physician-screened vs. unscreened individuals [21, 22]. These studies highlight the efficacy of strategies that target both patient- and physician-directed activation for optimal skin cancer screening, diagnosis, and management.

Despite the unique risk factors and extremely high rates of skin cancer among HCT survivors, less than 20% of survivors report having performed SSE within the past 2 months and received a physician examination for skin cancer in the past year [23]. These low rates of screening are compounded by the inconsistent recommendations for skin cancer screening in HCT survivors by national guidelines (e.g. National Comprehensive Cancer Network [no specific guidelines], American Society for Transplantation and Cellular Therapy [routine self-skin examination encouraged] [24]). Spurred by the large gap between rates of disease and practice, a recent NIH-sponsored initiative called for studies to address barriers to risk-based cancer screening after HCT, recommending survivor- and physician-directed interventions to increase awareness and early detection of suspected cancers [25]. In response, our study team has already developed and tested materials to enhance the practice of thorough SSE in HCT survivors and to encourage physician activation; we will use these materials in the proposed study.

### Gaps in HCT survivorship care and the importance of patient activation

We have shown that when long-term HCT survivors transition to PCPs, they no longer see their HCT physician on a regular basis, and rarely visit HCT survivorship care specialists [26, 27]. Thus, the long-term preventive care of HCT survivors is scattered across hundreds of thousands of individual PCPs. Although HCT survivors do seek primary care—more than 90% have seen their PCPs in the past two years—it is not in the cancer center context, where knowledge about the link between HCT-specific risk factors (e.g. radiation, GVHD) and skin cancer is more common [26, 27]. Therefore, it is of vital importance that HCT patients, who are at high risk of skin cancer, are trained to perform a thorough SSE and encouraged to ask their physician to conduct a thorough skin exam. Our research in the non-transplant setting indicates that PCPs are more likely to perform such exams when asked by patients to do so [28, 29].

Because skin cancer is the only cancer visible to the naked eye, photographs of suspect moles and lesions in print educational materials can boost recognition and practice of SSE [30]. Advances in technology, including the widespread availability of mobile phones and teledermoscopy (remote expert assessment of a photographed lesion) offer promising opportunities to improve early detection and treatment of skin cancer [31, 32]. The use of text messaging as an activation prompt is particularly appropriate because of the low cost, vast geographic coverage, and immediacy of this medium. In 2018, > 90% of the US population owned a mobile phone (with negligible differences by sex, age, education) [33]. To date, three randomized dermatology-related interventions have used mobile phones. These studies have consistently shown that mobile phone-based text messaging can improve skin cancer prevention and detection behaviors (e.g. sunscreen application) and self-screening rates, with high user satisfaction [34–36]. To induce patient activation, we will therefore distribute printed SSE educational materials to participants, followed by a series of tailored text messages to complement the mailed printed materials.

### The importance of physician activation to implement skin cancer screening

Although follow-up guidelines exist for HCT survivors, they do not explicitly state which provider (e.g. hematologist, PCP, other subspecialist) should manage which aspects of survivorship and preventive care. This lack of clarity for follow-up for survivors can result in under- or over-utilization of screening and diagnostic tests and add undue burden on the patient and care delivery system. These challenges are compounded by a lack of communication and coordination of care between oncology specialists and PCPs, as well as inadequate preparation of PCPs to deliver risk-based survivorship care [37]. These gaps in survivorship preventive care can be addressed through novel engagement strategies that bridge the gap between the specialized (e.g. HCT) providers and the PCPs who will ultimately be tasked with lifelong screening of at-risk survivors. Specific to this proposal, studies of PCPs have shown that they desire better training in dermatology and triaging cutaneous lesions, and that such training can be effectively provided across a variety of e-learning platforms [38, 39]. For the current proposal, we will build upon our past experiences with skin cancer screening studies in PCPs to deploy an HCT-focused e-learning module that informs PCPs about unique risk factors in HCT survivors and facilitates their completion of total body skin exams for these high-risk patients.

### Patient activation model

Our proposed survivor-directed intervention will be guided by the Patient Activation Model, which posits that an activated patient is better prepared to participate in self-management activities [40–43]. Patient activation is increasingly seen as central to achieving improved quality of care, better health outcomes, and less-costly health care utilization [42]. Patient activation involves four stages: 1) believing that taking an active role as a patient is important, 2) having the confidence and knowledge necessary to take action, 3) taking action to maintain and improve one’s health, and 4) staying the course even under stress [40]. The significance of patient activation has been recognized in ongoing health care reform efforts, including by the Center for Medicare and Medicaid Innovation [41, 42]. Since 2004, a number of studies have shown patient activation to be related to healthy behaviors (e.g. physical activity, healthy diet), appropriate use of the health care system (e.g. having a regular and timely source of care), and chronic care self-management (e.g. keeping a diary of blood pressure readings) [43–45]. Additional evidence suggests that PCPs likely play an important role in increasing patient activation. For example, one study found that PCPs who helped their patients in very concrete and specific ways (e.g. learning to monitor their condition, setting goals), had greater numbers of activated patients than those who did not [46]. Further, a checklist brought by a patient to a routine visit can serve as an activation tool for a provider and has been shown to encourage a PCP to: examine the patient’s skin, make a referral if necessary, motivate the patient to conduct SSE, record the high-risk status of the patient, and make early detection practices routine in subsequent visits [47]. As such, we will integrate a checklist as part of our patient-directed intervention.

### Physician-directed intervention

Our proposed physician-directed intervention is informed by the established patterns of long-term follow-up care after HCT [23, 26, 27], whereby a patient is transitioned from specialized cancer centers to the community, following stabilization of the patient’s acute medical needs. In this context, the community-based Shared-Care Model [37] allows optimal coordination between the HCT physician and other physician groups providing care. Simply stated, shared care refers to the care of a patient that is shared by two or more different specialties (or systems that are separated by some boundaries). The Shared-Care Model has been demonstrated to improve patient outcomes and enhance the management of patients with various chronic diseases including diabetes [48] and chronic renal disease [49], as well as those receiving oral anticoagulant therapy [50]. The cornerstone of shared care is communication and a period of transfer of knowledge between the specialist (e.g. oncologist/HCT physician) and the PCP.

### Leveraging technology to facilitate follow-up of abnormal findings

**Fig. 1:**
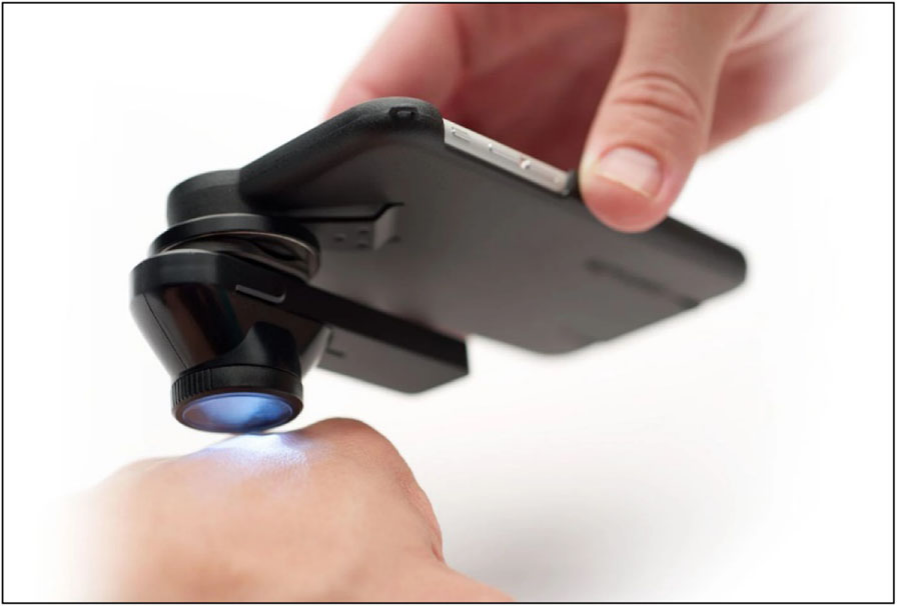
DermLite Dermatoscope. DermLite (www.dermlite.com) works with virtually any smartphone and tablet

For many patients and PCPs, lack of access to expert examinations and long wait times to see a dermatologist hinder/delay diagnosis and preclude treatment of early-stage skin cancers. A national survey of dermatologists found mean wait times for patients with an urgent changing mole was 38 days (range: 20–73) [51]. Teledermoscopy (TD) leverages the power of technology to enable clinicians to interact with dermatologists remotely, in less time. Acquiring dermoscopic images of lesions using a special magnifying lens allows key details of lesions to be transmitted to a dermatologist in real time (Fig. 1) [32]. We have shown that TD can improve the sensitivity and specificity of skin cancer detection by PCPs, and can identify smaller-diameter BCC, nearly all of which can be treated by PCPs using shave biopsy or the topical agent imiquimod, rather than the invasive surgical procedures required to treat larger lesions by a dermatologist [52]. As of 2018, there were > 60 TD centers in the US [53]; given the continued rapid expansion of technology and the emphasis on improving quality of care, the presence of TD in the US will likely continue to grow. For the current study, we will rely on well-established TD platforms that are rapidly scalable for the primary care setting, allowing for timely identification and referral of suspect lesions.

## Methods/design

**Fig. 2:**
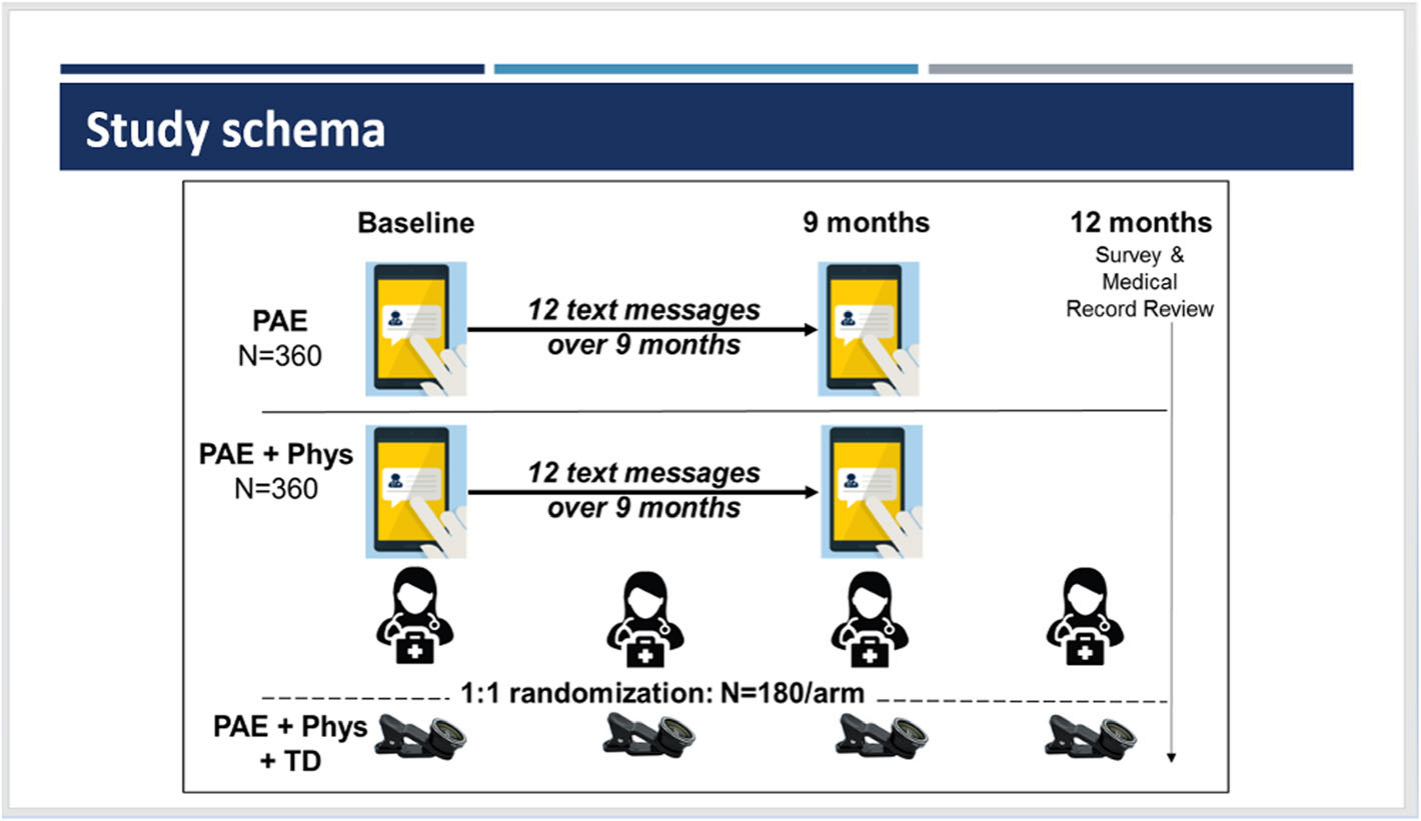
Study Schema for different arms. PAE: Patient Activation/Education (educational materials and text messages). PAE + Phys: Patient Activation/Education (educational materials and text messages) + physician activation/educational materials targeted to identified primary care providers (PCPs). PAE + Phys+TD: Phys: Patient Activation/Education (print materials and text messages) + physician activation/education + teledermoscopy (PCPs receive a dermatoscope to upload images of suspicious skin lesions to the remote study dermatologist)

In this randomized controlled trial, we will prospectively enroll 720 HCT patients over a three-year period, and will use a comparative effectiveness design (Fig. 2) comparing: *patient activation and education* (PAE, *N* = 360), which consists of patient-directed print materials and text messaging (12 messages over a 9 m period) vs. *PAE plus physician activation* (PAE + Phys, N = 360), which also includes physician-directed activation/educational materials about: 1) survivors’ increased skin cancer risk; 2) the benefits of and the skills needed to conduct full-body skin exams; and 3) the importance of recommending routine SSE to patients. Among the 360 survivors assigned to PAE + Phys, we will further randomize (1:1) the PCPs to receive either *printed physician activation materials* (*N* = 180) or *printed physician activation materials plus an e-learning module with access to TD* (PAE + Phys+TD; N = 180), which includes provision of a dermoscopic lens to take photographs of suspect lesions for review by the teledermatologist and recommendations and action steps needed to obtain expedited care for his/her patient. Participants and physicians will take part in the study for 12 m, with efficacy and endpoint assessments at the 12 m follow-up.

Inclusion requirements are as follows: have undergone autologous or allogeneic HCT at COH; are 1 to 5 years after HCT; ≥18 years of age at the time of enrollment; have seen a PCP in the previous 12 m or planning to do so in next 12 m; have a mobile phone with the ability to receive text messages; are able to read and write in English or Spanish; and are able to provide informed consent. Exclusion criteria are as follows: have no evidence of active hematologic malignancy or acute illness that would limit study participation.

### Participants and randomization

Eligible patients will be identified and recruited at COH from existing databases and physician referrals. Study staff will pre-screen everyone by reviewing the medical charts and will exclude anyone with conditions or reasons that may prohibit study entry. Participants who meet study eligibility criteria will be invited to enroll. All participants are enrolled on the study once they provided written consent and all eligibility requirements for the study have been met.

Participants are randomized 1:1 to PAE or PAE + Phys using a blocked stratified randomization. Stratification factors will include age (< 50, ≥50 years), sex (male, female), HCT type (autologous, allogeneic), and race/ethnicity (white, other). Participants randomized to PAE + Phys (*N* = 360) will be further randomized 1:1 to the PAE + Phy + TD arm, which includes the addition of an e-learning web-based skin cancer screening module with TD (*N* = 180) versus print materials alone (N = 180). To ensure that enrolled participants reflect the demographics of all eligible patients, we will stratify recruitment by age (+/−5y), sex, race/ethnicity, and HCT type (autologous/allogeneic). The current study has been approved by the Institutional Review Board of City of Hope (approval number: IRB #20096).

### Patient-directed intervention

All participants receive a study packet in either English or Spanish that includes: a personalized letter from the COH team welcoming them to the study; print materials about their skin cancer risk (highlighting HCT-specific risk factors), instructions on how to conduct SSE, pictures of worrisome lesions, an appointment checklist, and a checklist to help them maximize the effective of their physician skin examinations. Participants will then receive 12 separate text messages (once every 3 weeks) throughout the 9 m period designed to encourage them to:

- Thoroughly examine their skin using the instructional pictorial diagrams and photographs of abnormal lesions provided in the print materials;
- Request physician skin exams and bring the checklist to their PCP; and
- Develop a collaborative care plan (between the PCP and participant) including common responsibility for monitoring and quickly following up on new and changing moles and lesions.

The specific patient baseline and 12 m measures are summarized in Table 1. To optimize patient participation, we will employ proven strategies that include:

- Providing electronic and paper questionnaire options,
- Offering incentives ($40/set of completed questionnaires, baseline and 12 m), and
- Sending mailed reminder notices and text messages ahead of the 12 m questionnaire.

**Table 1:** Measures and definitions

M**easure**	Definition
**SkinSelf-Examination** (SSE)	Defined as performing at least one SSE during the 2 m prior to the baseline and 12 m follow-up assessments [54–56]. Additionally, survivors will be asked how often in the prior 2 m they had thoroughly examined each of eight areas of the body (“the front of you from the waist up,” “the front of your thighs and legs,” “the bottom of your feet,” “your calves,” “the backs of your thighs,” “your buttocks and lower parts of your back,” “your upper back,” and “your scalp.”) This serves as a measure of quality of each patient’s report of the SSE.
**PhysicianSkinExam**	The physician skin exam will be based on response to the question, “During the past 12 months, has a doctor deliberately checked all or nearly all of your whole body for the early signs of skin cancer?” We will ask about the extent of the examination (“Did it include the lower back? […] The scalp?”) and whether the participant was completely undressed for any part of the examination [54–56]. Patients will be asked whether the examination took place during a scheduled visit or was prompted by the participant’s finding of a new lesion
**SkinCancerDetectionKnowledge**	Skin Cancer Detection Knowledge will be assessed using an established questionnaire on skin cancer risk factors (e.g. skin phototype, family history of skin cancer) [57].

### Physician-directed intervention

At study enrollment, participants will be asked to provide the name and contact information of their PCP and ideally the date of their next PCP visit. The physicians of patients randomized to PAE + Phys will receive a packet consisting of:

- A letter that describes the intervention and encourages them to do a full-body skin examination at the patient’s next visit and to recommend that the patient performs SSE;
- Information about HCT survivors’ increased risk of skin cancer;
- Images of suspect moles and lesions; and
- Instructions on how to perform a full-body skin examination.

Per the Shared-Care Model of Survivorship Care, the top sheet of the packet will include a personalized letter by from the patient’s HCT physician reminding the PCP about the risk of skin cancer among HCT patients and encouraging them to screen for suspicious moles and lesions. We will send the physician activation materials approximately 7 days after patient enrollment and a reminder letter approximately 10 days before the patient visit. This approach ensures that physicians have the activation materials in case they receive participant inquiries prior to the scheduled appointment.

Physicians of patients randomized to PAE + Phys+TD will receive the same educational materials as PAE + Phys. In addition, they will receive a free portable dermatoscope with instructions for uploading images of suspect lesions. The dermatoscope can be attached to smartphones and tablets and allows for high-quality visualization of subsurface skin structures that are not usually visible to the naked eye. Advantages of this technology are that it can facilitate detection of skin cancers in the early stages of development. Physicians in the PAE + Phys+TD will also be asked to view an online module that will provide clear instructions for using a dermatoscope, and steps to integrate dermoscopy into their practice. Physicians will be strongly encouraged to take pictures of lesions of patients for whom they have a low to moderate level of concern of malignancy. The study dermatologist will review all dermoscopic images and provide written feedback to PCPs within approximately one week. Report outcomes will range from:

- Image of insufficient quality, photograph needs to be re-taken, with instructions on how to improve resolution;
- Image of sufficient quality–benign;
- Image of sufficient quality–benign, but final management decision will rest on re-evaluating a repeat image taken four weeks later; and
- Image of sufficient quality–lesion of concern and participant instructed to see a dermatologist.

We expect that teledermoscopy reports revealing positive findings will spur physicians and patients to obtain expedited follow-up care, including speedier referrals to dermatologists, thereby reducing wait times.

### Statistical considerations

We will test our first hypothesis that compared to PAE, participants randomized to PAE + Phys will report higher rates of thorough SSE *and* physician skin exam. We will administer patient questionnaires at baseline and 12 m to ask about skin examinations performed within the past 12 months. We will generate a binary outcome (Y) based on patient responses from the 12 m end-of-study questionnaire, whereby Y = 1 if they report having conducted a SSE and having received a physician skin examination. All other responses will be considered Y = 0. Due to randomization, we do not expect differences in baseline patient and treatment characteristics between the two study arms. Nevertheless, we will compare baseline characteristics (e.g. age, sex, race/ethnicity, diagnosis, type of HCT, radiation exposure, severity and extent of GVHD) of the two groups using standard univariate analyses. To determine the efficacy of the intervention, we will conduct intention-to-treat analysis. We will compare the proportion of patients who report having conducted an SSE *and* had a physician exam during the 2 m prior to the 12 m follow-up assessment, using Chi-squared analysis. We will use logistic regression to adjust for imbalance in patient characteristics and risk factors. We will also test for group by covariate interactions, depending on group main effect. Per the ASK trial [58], we anticipate the proportion (*p)* of patients who report SSE *and* a physician exam during the 2 m prior to the 12 m follow-up assessment in the PAE arm to be 40%.

Assuming a Type I error = 5%, 270 patients per arm at month 12 (accounting for 25% attrition from baseline) will provide 80% power to detect a minimum percentage point difference of 12% or an odds ratio of 1.62 between the two study arms (Table 2). Our previous experience with patient- and PCP-directed interventions suggests that a 12% difference is a conservative estimate and achievable for the current study.

**Table 2:** Projected proportions of patients who will report SSE and PCP exam at 12 m (PAE vs. PAE + Phys)

PAE (proportion [p1])	40%	40%	40%
PAE + Phys (p2)	52%	55%	60%
Odds ratio	1.62	1.83	2.25
Number (per arm)	270	173	97

In addition, we will test our second hypothesis that compared to PAE, participants randomized to PAE + Phys will report shorter time to definitive diagnosis of suspect lesions. From data reported on the patient’s 12-month questionnaire, we will determine the time interval between a participant’s first notice of a suspect mole or lesion and the date on which a definitive diagnosis was made (i.e. by the PCP or a dermatologist). The outcome will be a continuous variable and we will employ a generalized linear model (GLM) to compare the interval between the two study arms, adjusted for covariates of interest. We will begin with bivariate models to determine potential variables to include in a multivariable regression model. If the group main effect is significant, interactions of the group main effect with other variables will be examined. Power calculation: We will assume that 10 to 30% of patients will have a concerning mole or lesion for which they will seek care from their PCP or a dermatologist during the study period [12, 59, 60]. Mean wait time (time between call for appointment and definitive diagnosis by their PCP or a dermatologist, if equivocal) was assumed to be 30 days [51], with a range of standard deviations (SD = 5 to 15). Based on these values, we will have 80% power at Type I error = 5% to detect a significant group difference ranging from 1.9 days (SD = 5) to 5.7 days (SD = 15) if 30% of participants seek follow-up care; or 3.3 days (SD = 5) to 10.0 days (SD = 15) if only 10% of participants seek care for a concerning mole or lesion by 12 m (Table 3).

**Table 3:** Detectable difference in time to definitive diagnosis between arms, given a range of proportions (patients with concerning lesions), and standard deviations (SD) around mean time

Proportions	SD	Group Difference
10%	5	3.3
	10	6.7
	15	10
20%	5	2.4
	10	4.7
	15	7.1
30%	5	1.9
	10	3.8
	15	5.7

Finally, we will test our hypothesis that PCPs randomized to PAE + Phys+TD will have greater recognition of suspect lesions and more appropriate as well as cost-effective referral patterns compared to PAE + Phys alone. We will administer questionnaires at baseline and 12 m to all PCPs of participating patients to assess their ability to *recognize* suspect lesions and their self-confidence in performing the skin cancer exam and making referrals. We will compare the group difference in changes in attitude over time, using GEE for normally distributed data, with a compound symmetry covariance matrix analysis to account for within-physician correlation. We will dichotomize the Likert scale [1–5] response and compare the proportion of PCPs reporting a higher (≥4 vs. < 4) level of confidence at 12 m compared to baseline between groups, using the longitudinal binomial GEE model with a compound symmetry covariance structure. Covariate adjustment will be made in these models as necessary. *Power Calculation:* With 180 PCPs per arm at baseline and 135 at 12 m, assuming a Type I error = 5%, we will have 80% power to detect odds ratios ranging from 2.7 to 4.0 (corresponding to absolute differences: 20–30%) at 12 m, given a participation rate range of 50–70%.

### Sample size calculations

We will approach approximately 850 potentially eligible patients for enrollment; we anticipate 720 (85%) patients will consent and be randomized (360/arm) at baseline, and 540 (75% retention) will be evaluable at 12 m (270/arm).

### Economic impact measures

To determine intervention cost estimates, we will include both the cost of intervention components as well as the cost of implementing the intervention. Intervention component costs include print materials and mailing, text messages, dermatoscope, and personnel time. Time spent developing materials will not be included, as these are fixed, and would not be incurred if another site adopts the intervention. We will value commercially available items, such as dermatoscopes, at their average retail price rather than the subsidized rate provided specifically for this study. We will collect information for estimating intervention costs from study records and personnel reports. We will value personnel time at prevailing national average wage rates for the relevant occupational categories and explore alternative values in sensitivity analysis.

To estimate the economic impact of the intervention, we will survey patients at the 12 m assessment regarding their use of specific health care services. They will be asked about visits with PCPs and dermatologists, receipt of relevant diagnostic procedures including biopsies and imaging, and treatment for any newly diagnosed skin conditions. Each health care service will be multiplied by a unit cost amount in order to estimate total costs per participant. We will use Medicare’s Direct Practice Expense and Resource-Based Relative Value Scale to estimate average unit costs for physician and laboratory services. Although some study participants will not be Medicare beneficiaries, Medicare’s reimbursement methodology was developed to reflect true resource costs. For this reason, Medicare reimbursement may be used as a proxy for unit cost, even when the population of interest is not limited to Medicare beneficiaries. This methodology has been employed in economic analyses of other cancer screening interventions. In sensitivity analysis, we will evaluate a range of unit cost estimates [61, 62].

Our assessment of the downstream costs of the intervention, as well as the cost of the intervention itself, will allow us to perform a limited cost-effectiveness analysis. Specifically, we will estimate the cost per additional SSE completed and the cost per additional PCP exam completed, comparing the two intervention arms. Given that the primary focus of the trial on non-economic endpoints and the associated sample size requirements, we will not conduct formal hypothesis testing on the economic outcomes. The economic impact of the intervention will be evaluated using standard incremental cost-effectiveness analysis methods, and sensitivity analysis will be used to assess the impact of assumptions and uncertainty on results and conclusions. Although estimation of lifetime costs and outcomes associated with the study interventions are beyond the scope of this proposal, results of the cost analyses will serve as preliminary data for future interventions that explore the long-term cost-effectiveness of increasing skin cancer screening among HCT survivors.

## Discussion

A key factor to consider when developing any preventive health intervention is sustainability, and the likelihood of dissemination outside of the research setting should the intervention be found effective (scalability). Health care settings, in particular, pose real challenges to implementation of behavioral interventions, due to the time and skills needed for implementation. Interactive technologies based on evidence-based behavior change principles can begin to address barriers to implementation of behavior change programs in health care.

These technology-enabled interventions can ensure that a consistent, high-quality message is delivered to patients, can inform patient–provider interactions, and can maximize staffing efficiencies; they can also address a wide variety of behaviors simultaneously. Thus, interactive technology can provide a streamlined, consistent method for conducting many aspects of evidence-based behavior change counseling. Randomized controlled studies have demonstrated that use of interactive technologies does support lifestyle behavior change in patients.

Studies of PCPs have shown that they desire better training in dermatology and triaging cutaneous lesions, and that such training can be effectively provided across a variety of e-learning and technology-enabled platforms. We have designed this intervention with careful consideration of how it might be sustained outside of the research context. Delivery of the PAE intervention can easily be integrated into HCT care, as it is routine to provide patients with written or electronic materials related to ongoing care. If the intervention is effective, we envision working with national organizations (e.g. American Cancer Society; American Society for Transplantation and Cellular Therapy) to determine the best strategies for dissemination to HCT programs throughout the country.

## Conclusion

HCT survivors have been exposed to cumulative therapeutic exposures that injure normal tissues, leading to premature onset of chronic health conditions such as subsequent cancers [6, 7]. HCT survivors have a > 10-fold risk of developing subsequent cancers after HCT [8, 9], with skin cancer accounting for up to 60% of all subsequent solid cancers [8, 10]. Importantly, the epidemiology of skin cancer in HCT survivors differs from that of the general population, as survivors develop skin cancers at a younger age, present with advanced disease, and are more likely to have multiple recurrences, with a shorter latency between recurrences [11, 12, 17, 18]. Despite the unique risk factors and extremely high rates of skin cancer among HCT survivors, less than 20% of survivors report having performed skin self-exam (SSE) within the past 2 months and received a physician examination for skin cancer in the past year [23].

In HCT survivors, skin cancers develop at a time when their follow-up has largely transitioned to the primary care setting [12], emphasizing the need to develop innovative strategies that educate survivors and PCPs alike to improve skin cancer self-examination and clinical screening rates. Thus, we have developed a 12 m intervention focused on early detection of skin cancer and timely medical follow up among HCT survivors. Findings from this study can 1) establish the efficacy of PAE, and the relative benefit of physician activation; 2) inform the practice of skin cancer screening using innovative strategies that are readily applicable in the clinical setting; and 3) identify facilitators of and barriers to appropriate delivery of survivorship care in long-term HCT survivors. It is anticipated that results obtained from this intervention can potentially help develop strategies to facilitate the screening and management of other late effects (e.g. cardiovascular, endocrine) in HCT survivors, and to improve skin examination vigilance and skin cancer detection in other high-risk (e.g. radiation-exposed) cancer survivor populations. Given the emerging interest in remote patient monitoring and healthcare delivery due global crises such as the COVID-19 pandemic, it is especially timely to consider new paradigms to address the needs of cancer survivors.

## Trial status

Study enrollment has not commenced.

## Data Availability

Study enrollment has not yet commenced; thus, there is no available data or materials.

## Abbreviations

ABCDE: Asymmetry, Border, Color, Diameter, Evolution
BCC: Basal cell carcinoma
COH: City of Hope
GVHD: Graft-versus-host disease
HCT: Hematopoietic cell transplantation
ICF: Informed consent form
m: month
NMSC: Non-melanoma skin cancer
PAE: Patient activation and education
PAE + Phys: Patient activation and education plus physician activation
PAE + Phys+TD: PAE + Phys plus e-learning web-based skin cancer screening module with teledermoscopy
PCP: Primary care physician
SCC: Squamous cell carcinoma
SD: Standard deviation
SSE: Skin self-examination
TD: Teledermoscopy
US: United States
USPSTF: U.S. Preventative Services Task Force
